# The Impact of Lifestyle on the Secondary Sex Ratio: A Review

**DOI:** 10.3390/life14060662

**Published:** 2024-05-22

**Authors:** Iasonas Dermitzakis, Paschalis Theotokis, Evangelos Axarloglou, Efthymia Delilampou, Dimosthenis Miliaras, Soultana Meditskou, Maria Eleni Manthou

**Affiliations:** Department of Histology-Embryology, School of Medicine, Aristotle University of Thessaloniki, 54124 Thessaloniki, Greece; iasonasd@auth.gr (I.D.); ptheotokis@auth.gr (P.T.); evanaxar@auth.gr (E.A.); edelilamp@auth.gr (E.D.); miliaras@auth.gr (D.M.); sefthym@auth.gr (S.M.)

**Keywords:** lifestyle, secondary sex ratio, diet, psychological distress, socioeconomics, occupation, reproduction, development

## Abstract

The secondary sex ratio (SSR), indicating the ratio of male to female live births, has garnered considerable attention within the realms of reproductive biology and public health. Numerous factors have been posited as potential trendsetters of the SSR. Given the extensive research on the impact of daily behaviors and habits on individuals’ reproductive health, there is a plausible suggestion that lifestyle choices may also influence the SSR. By synthesizing the existing literature on the current research field, this comprehensive review indicates that an elevated SSR has been associated with an increased intake of fatty acids and monosaccharides, proper nutrition, higher educational levels, financial prosperity, and favorable housing conditions. On the other hand, a decreased SSR may be linked to undernutrition, socioeconomic disparities, and psychological distress, aligning with the Trivers–Willard hypothesis. Occupational factors, smoking habits, and cultural beliefs could also contribute to trends in the SSR. Our review underscores the significance of considering the aforementioned factors in studies examining the SSR and emphasizes the necessity for further research to unravel the mechanisms underpinning these connections. A more profound comprehension of SSR alterations due to lifestyle holds the potential to adequately develop public health interventions and healthcare strategies to enhance reproductive health and overall well-being.

## 1. Introduction

The secondary sex ratio (SSR), defined as the ratio of male to female live births, has long been a topic of interest in various fields, including reproductive biology, public health, and social sciences [1]. The worldwide average SSR is approximately equal to 1.05, indicating a slight predominance of male newborns over females [2]. Although the determination of the SSR and overall embryonic development was previously believed to be exclusively governed by intrinsic stimuli, contemporary research has revealed that external factors may also contribute to the modulation of these processes [3,4,5]. Specifically, seasonal variations, terrorism, wars, exposure to chemicals, infections, natural disasters, and nuclear accidents have all been recognized as potential trendsetters of the SSR [6,7,8,9,10,11]. Considering the effects of daily behaviors and habits on individuals’ reproductive health, there is a viable hypothesis that lifestyle choices may also influence the SSR [12].

The impact of lifestyle on the SSR is a complex and multifaceted subject that necessitates a comprehensive understanding of the interplay among various environmental, social, and individual factors. Dietary habits, calorie intake, socioeconomic conditions, psychological stress, and occupation have been proposed to affect the SSR [13,14,15,16,17]. Research indicates that diets high in calories and specific nutrients have been linked to changes in reproductive outcomes, potentially influencing the odds of conceiving male or female offspring [18]. Moreover, socioeconomic status, which encompasses income, education, and resource accessibility, significantly influences overall health and well-being across populations [19]. Studies demonstrate that socioeconomic disparities can impact reproductive behaviors, fertility rates, and birth outcomes, thereby potentially affecting the SSR [20]. Psychological stress is also a crucial aspect of lifestyle that is associated with modifications in biological processes and reproductive health [21]. Along the same line, occupation, as a fundamental aspect of lifestyle, can additionally mold reproductive patterns and outcomes by influencing work-related stress, physical demands, and environmental exposures. Certain occupations may involve elevated stress levels, irregular working hours, or exposure to hazardous materials, which can affect reproductive health and fertility [22].

Fluctuations in the SSR may be engendered by factors that act either during conception or throughout gestation [23]. Initially, the determination of the sexes of zygotes, serving as the basis for calculating the primary sex ratio, is purportedly influenced to some extent by the hormonal levels of both parents during the peri-conceptional period [24]. It is hypothesized that elevated concentrations of sex-specific parental hormones, such as estrogen and testosterone, are correlated with the birth of male offspring. At the same time, heightened levels of progesterone and gonadotrophins are linked to the birth of female offspring [25]. The established sex ratio at conception undergoes additional modifications as a result of male-biased spontaneous abortions induced by high levels of adrenal androgens; additionally, the increased secretion of androgens is attributed to maternal stress. This ultimately contributes to the observed sex ratio of offspring at birth [24]. An evolutionary theory also has a prominent role in interpreting fluctuations in the SSR, namely the Trivers–Willard hypothesis. It postulates that parents in “good conditions” are more likely to have offspring of the male sex in order to maximize reproductive success [26,27].

The present review aims to investigate the complex relationship between lifestyle factors and the SSR. Specifically, we analyzed and summarized the existing literature to elucidate how lifestyle, including diet habits, calorie intake, socioeconomic conditions, psychological stress, and occupation, may influence the proportion of male-to-female births in populations. By synthesizing the evidence and exploring the potential mechanisms underlying these associations, this review seeks to provide insights that may have implications for reproductive and public health.

## 2. Review Methodology

The current review aims to examine the impact of lifestyle on the SSR using a structured methodology. While the review is not systematic, specific procedures were adhered to. A search was carried out across prominent academic databases, including PubMed, Scopus, and Web of Science, utilizing keywords such as “secondary sex ratio”, “sex ratio at birth”, “diet”, “calorie”, “socioeconomic”, “education”, “stress”, “occupation”, “smoking”, “culture”, “primary sex ratio”, “sex ratio at conception”, and related terms. All relevant papers from the inception of each database were considered for analysis, and the reference lists of these publications were reviewed for additional sources. The inclusion criteria comprised peer-reviewed original articles and reviews published in English with full-text availability. Publications meeting the criteria underwent screening based on titles and abstracts to determine their eligibility. Following this, relevant data, including the study design, factors under investigation, and findings on SSR variations, were extracted from the selected studies for an in-depth analysis. A narrative synthesis method was employed to summarize and interpret the primary findings from the referenced literature.

## 3. Dietary Habits and Calorie Intake

The Western diet model, characterized by the consumption of refined foods and a high intake of simple sugars and salt, has been identified as a significant contributing factor to various pathological conditions, including cardiovascular disease, chronic inflammation, and type 2 diabetes mellitus [28,29,30]. Since the early 2000s, several studies have investigated the impact of dietary components on the SSR, initially focusing on mice and other mammals.

### 3.1. Dietary Habits and SSR

#### 3.1.1. Fatty Acids

A higher fat percentage in the diet seems to skew the SSR in favor of males [18,31,32]. Initial studies in mice provided data supporting that a diet that is higher in fatty acids (i.e., 60%), saturated or not, results in more male offspring than a diet with a smaller percentage of fatty acids (i.e., 10%), even if they both contain similar daily calorie intake [31]. Specifically, Rosenfeld et al. conducted a study using mice that were fed either a very high or low saturated fat diet from 30 days old until the mice had successfully weaned their first litters of pups. The SSR was statistically significantly higher in the high-fat diet group. Conversely, a diet composed of less fatty acids and more carbohydrates led to a female-skewed SSR. Such findings are explained by the Trivers–Willard hypothesis [27]. According to that theory, mothers with “good conditions”, which relate to a high-fat diet in this study, are more likely to produce male offspring.

A study conducted in 2018 shed light on the preconception effect of a maternal high-fat diet on the SSR [13]. The experiment was carried out on a private farm at Mansoura University, and Holstein cows were used in late lactation. The animals were divided into three groups. The first group received a regular diet, while the second and third groups were supplemented with 3% and 5% protected fat, respectively. Embryos formed through in vitro fertilization (IVF) were then implanted in the cows. The oocytes used for the IVF procedure were matured with one of four solutions, namely omega-3 alpha-linolenic acid (ALA), omega-6 linoleic acid (LA), trans-10, or cis-12 conjugated linoleic acid (CLA), or a control solution. The findings suggest a statistically significant increase in the SSR in the groups where the diet was supplemented with fatty acids. Regarding the maturation of oocytes with the four different solutions, the SSR increased significantly after maturation with LA or CLA. In contrast, maturation with ALA or the control solution (without any added fatty acids) did not show a difference in the SSR. The Trivers–Willard hypothesis also explains these findings, as the SSR upshifted in accordance with an increase in fatty acids. Furthermore, the maturation of oocytes with polyunsaturated fats led to a male offspring excess compared to the control solution. This suggests that fatty acids may be incorporated into the cellular structure of the oocyte, potentially altering its adhesion and signaling molecules and thereby favoring male implantation.

#### 3.1.2. Sugar Intake

Regarding the intake of simple sugars, increased glucose levels have been shown to skew the SSR towards male embryos in a dose-dependent manner [33]. Specifically, when bovine blastocysts at the eight-cell stage were exposed to different glucose concentrations in vitro, there was a selective reduction in the development of female blastocysts in the uterus. This pathophysiological pathway is likely related to the pentose phosphate pathway, which is four times more active in female bovines than in males, potentially due to the X-chromosome loci of the G6PD gene [34,35]. Although the pentose phosphate pathway is typically considered protective against free radical damage, it is noteworthy that one or more metabolites (e.g., NADPH) produced through the enhanced activation of this pathway may potentially disrupt fetal development [36,37,38,39,40,41]. In line with these findings, Cameron et al. investigated the effect of the glucose concentration on primiparous mice [42]. The mice were divided into two subgroups: a control group and a group exposed to dexamethasone added to drinking water during the mating phase. This steroid inhibits glucose transport and reduces plasma glucose concentrations [43]. After three days, the dexamethasone water was replaced with clear water, limiting the exposure to the early cell division stage. The results revealed a statistically significant skew towards female births in the dexamethasone-exposed group compared to the control group. A model selection analysis indicated that the difference in glucose concentration was the causative factor, suggesting a potential pathophysiological pathway involving the selective loss of early cell division male conceptuses in the uterus.

Furthermore, fructose levels also positively affect the SSR, but through different pathways. Increased fructose consumption leads to elevated levels of glucose, triacylglycerols, and cholesterol in maternal blood [44]. Although the metabolic consequences of excessive fructose intake are well established, no toxic effects were observed in the experiment with bovine blastocysts mentioned above at a concentration of 5.6 mΜ [33,45]. More recently, an experiment involving rats investigated the effects of consuming a 10% fructose solution or a 4% NaCl solution on the SSR [44]. The rat population was divided into four subgroups. It was noted that the groups with 10% fructose added to their water showed an increase in the SSR. The potential effect of a high-salt diet was also examined, but no statistically significant skew in the sex ratio was observed. This observation aligns with the Trivers–Willard hypothesis. The skew in the SSR appears to be a maternal matter, as the exposure of male rat spermatozoa to a high-fructose solution did not significantly affect their motility. There is a high possibility of a preconception effect on female rats’ oocytes. Selection in utero is less likely, as there were no differences between the sex ratio at conception and the SSR.

Based on the available data, elevated fatty acid and monosaccharide intake is associated with an increased SSR. This correlation aligns with the Trivers–Willard hypothesis. However, these data have to be confirmed and extensively investigated in human models to derive precise and reliable conclusions.

### 3.2. Calorie Intake and SSR

The maternal weight could be considered an index of the mother’s general health status and reflects the quality of nutrition and caloric intake. Studies in this field have stressed the impact of the maternal diet on fetal development and intrauterine growth. For example, insufficient weight gain during gestation has been associated with low-birthweight offspring, which are more prone to cardiovascular and metabolic diseases. Other than that, gestational weight gain beyond normal limits has been blamed for certain adverse birth outcomes, namely preterm birth and emergency cesarean delivery [46,47].

There are several studies examining the relationship between maternal calorie intake and the SSR. In particular, an experimental research study was conducted on 72 adult female mice distributed into three sets according to their diet regimens: premating-deprived mice, gestation-deprived mice, and mice that were fed ad libitum [48]. Mice belonging to the premating-deprived category were intermittently fed for one week before mating. During gestation, they were administered the diet of the controls, which were fed ad libitum. The gestation-deprived mice only followed an intermittent dietary regimen during pregnancy. A statistical analysis revealed that the gestation-deprived and premating-deprived mice delivered lower percentages of males than the control mice. The results match the findings of a prior research study revealing that undernourished female rodents had a higher chance of delivering female neonates [49,50,51]. This evidence is in total agreement with the Trivers–Willard hypothesis.

In a retrospective study in Modena, Italy, researchers collected data for 9284 births, along with other parameters, such as the maternal body weight before conception and throughout gestation [14]. It was shown that mothers ranked in the lowest quartile of preconception body weight had a significantly lower SSR compared with the remainder of the study population. In contrast, higher weight gain during pregnancy induced a downward index shift. The SSR among women of the highest quartile of weight gain was 0.493, whereas the SSR of the first three quartiles combined was 0.516. It was speculated that women with higher weight gain during gestation may tend to store rather than use energy to sustain the fetus, thus leading to suboptimal pregnancy conditions, which jeopardize the viability of male embryos. Furthermore, the impact of maternal eating disorders on the SSR was investigated within a large population-based cohort comprising 38,340 pregnant women in Norway [52]. The analysis considered confounding variables such as the mother’s age, educational level, pre-pregnancy BMI, gestational age, smoking history, income, marital status, and parity. Lower proportions of male offspring were noted among women with anorexia and bulimia. In contrast, higher proportions of male births were linked with binge eating disorder and eating disorders not otherwise specified, such as purging-type eating disorders.

A recent study obtained records from 740 British women who were unaware of the sexes of their fetuses to establish a correlation between maternal dietary patterns at the time of conception and fetal sex [53]. Women with the highest pre-conceptional energy intake gave birth to more boys. However, calorie intake during pregnancy did not exhibit an association with fetal sex. These findings are consistent with hypotheses proposing a bias towards investing in male offspring during periods of resource abundance, as postulated by the Trivers–Willard hypothesis. Nevertheless, some studies that examined the effect of the 1944–1945 Dutch Hunger Winter on the SSR did not show an association between maternal undernutrition and a female-biased SSR [54,55]. A further analysis of the SSR trend was conducted using the birth records of 310,101 Chinese women [56]. It revealed a notable decrease in the SSR between April 1960, a year after the onset of the Great Leap Forward Famine, and October 1963, approximately two years after the famine termination. The inconsistent outcomes reported in earlier studies centered on the 1944–1945 Dutch Hunger Winter could be attributed to the brief duration of this famine, limiting its impact on sex ratio dynamics.

**Figure 1:**
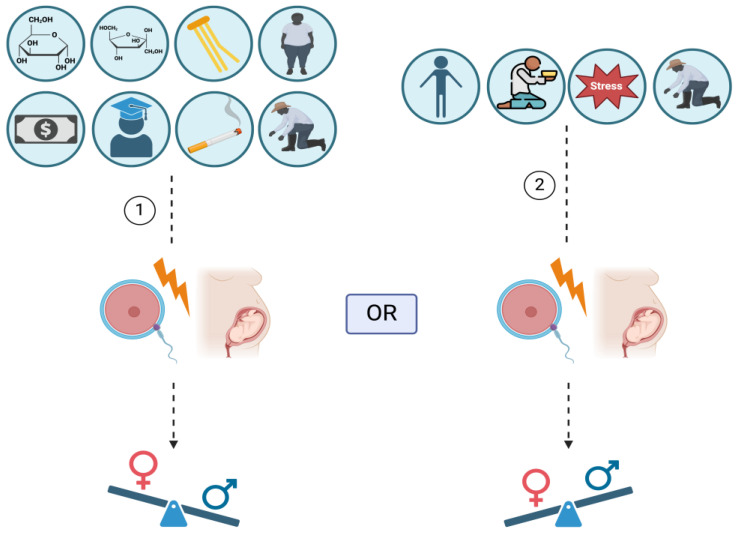
The impact of lifestyle on the secondary sex ratio (SSR). A diet rich in glucose, fructose, and fatty acids; optimal nutrition; socioeconomic prosperity; high educational attainment; smoking; and certain occupations have been associated with an elevated SSR (1). Conversely, undernutrition, economic disparities, psychological distress, and other occupations have been linked to a reduced SSR (2). The figure was created on BioRender.com.

All of the studies have demonstrated that a low pre-pregnancy maternal weight, as well as food deprivation during pregnancy, causes a downward shift in the SSR (Figure 1). Generally, male fetus pregnancies are more energy-demanding; thus, they cannot be sustained if the mothers are in a caloric deficit. It is speculated that this female-biased SSR is caused by a selection of gametes during fertilization and an elevated risk of male fetal losses due to suboptimal gestational conditions [14,48]. 

## 4. Socioeconomic Conditions

Socioeconomic conditions, such as income, residence ownership, and educational level, significantly impact the quality of people’s lives [19]. It is common knowledge that economic destabilization is a major stressor for communities affected and could thus be associated with changes in the SSR [57]. Several studies support this statement. Initially, a comparative study conducted in Israel over 15 years (1980–1995) examined the SSR within both the Jewish and Muslim populations [58]. Annual SSR records were collected and analyzed. The study suggested that socioeconomic factors, such as monthly income and housing density (i.e., the number of people per room), may influence psychological distress. The most affected group, namely the Muslim group, demonstrated a female-biased SSR. Additionally, the same research proposed that higher levels of maternal and paternal education had a positive impact on the SSR. A significant portion of Muslim communities displayed extremely low levels of education (i.e., less than five years of schooling). The effects on the SSR were less pronounced among Jewish individuals, who had an average education level of 9 to 12 years.

Economic stability at the societal level is also crucial. The fluctuation in the SSR was investigated in East and West Germany during the post-World War II period [20]. Following the country’s reunification in October 1990, the eastern part experienced economic difficulties, which had a notable impact on the SSR. The lowest point for the SSR in East Germany was observed in 1991. Subsequently, the SSR gradually recovered, supporting the hypothesis of a stress-induced reduction in male offspring due to poor sperm motility, the disruption of hormones, and changes in coital frequency. Thus, the pre-conceptional and in utero processes may have contributed to the observed decline together. In like manner, the increased occurrence of economic crises in modern societies impacts collective distress [59,60]. Various socioeconomic changes in different countries have temporarily manifested in the SSR. For example, the significant decline in the gross domestic product experienced in Japan in 1974 resulted in a noticeable decrease in the SSR one year later [61]. Similarly, the fluctuation in the SSR in Ireland reflected their economic instability. The overall higher SSR in Northern Ireland, in comparison to the Republic of Ireland, aligns with the economic stress theory, indicating that the comparatively less prosperous southern region exhibited a lower SSR than the more affluent northern region [62].

At the same time, wealth, which is generally considered an indicator of good health, positively influences the SSR [63]. An Australian study examined data from a nationwide sample of 5107 children born between 2003 and 2004, focusing on the SSR and socioeconomic conditions such as maternal education and wealth [15]. The SSR, defined as the ratio of males to total births, varied depending on the reported wealth of the mothers surveyed, ranging from 0.4069 to 0.5292 for women who reported poor and prosperous economic conditions, respectively. Furthermore, a demographic study of the SSR in South Korea during the 1981–2004 period found that maternal socioeconomic status was a more sensitive indicator of the SSR than paternal status [64]. The lack of residence ownership can also be a stressor and impact the SSR. An observational study was conducted in Uganda involving 500,000 families [65]. The study revealed a significantly higher SSR in families who owned dwellings than those without property. It is believed that mothers who lack permanent residence may experience higher levels of stress, leading to the production of glucocorticoids during pregnancy [66]. This, in turn, might put male fetuses in a more vulnerable condition [67].

Changes in women’s educational levels, particularly over the past century, have significantly and positively impacted their overall well-being. While limited data regarding its influence on the SSR are available in the literature, noteworthy findings have been reported. A comparative study was conducted examining the SSR of offspring born from important female personalities whose biographies were documented [68]. The results indicate a significantly higher SSR among these offspring than the general population. This observation aligns with the maternal dominance hypothesis, which suggests that women with higher dominance are more likely to have male offspring [69,70]. Supporting this notion, Almond and Edlund’s research study in the United States involved analyzing 48 million births from 1983 to 2001 [71]. Their findings revealed a positive correlation between high maternal educational levels and the SSR. These findings support the viability of the Trivers–Willard hypothesis, which proposes that evolutionary mechanisms favor deviations from the population sex ratio in response to parental conditions, particularly in modern societies [27].

In conclusion, socioeconomic conditions play a significant role in influencing the SSR (Figure 1). Factors such as wealth and adequate housing conditions positively impact the SSR. Additionally, as maternal educational levels elevate, the SSR ratio increases. These observations can be seen as an application of the Trivers–Willard hypothesis. Conversely, the SSR tends to decrease during periods of economic crises. Economic instability increases stress levels within the affected population, resulting in higher maternal glucocorticoid excretion. This, in turn, leads to the selected-in utero pathway acting against the more vulnerable male fetuses.

## 5. Psychological Distress

Living organisms receive a plethora of intrinsic or extrinsic stimuli and aim to adapt to them via stress responses. Excess stress can have a negative impact on cognition and brain structure as well as the immune, cardiovascular, and gastrointestinal systems [21]. Moreover, oxidative stress, through the elevation of reactive oxygen species, can lead to male infertility, interfere with ovulation, and cause fetal loss via placenta malfunction [72,73]. Therefore, stress phenotypes could potentially influence the SSR, opening up an intriguing avenue for further investigation.

A study set in Sweden aimed to elucidate whether there was an association between anxiety disorder prevalence and the SSR [16]. The incidence of anxiety disorders was monitored via records of doses of antidepressants and anxiolytics dispensed to women over 276 months. A statistical analysis revealed that the daily doses of this category of drugs demonstrated a negative correlation with the SSR. No significance in the relationship between the drug doses dispensed to men and the SSR was noted. Thus, the authors concluded that higher doses of antidepressants and anxiolytics, which translate to a higher anxiety disorder prevalence, were inversely associated with the SSR and may be attributed to increased male fetus loss driven by stress. Subsequently, a case–control comparative study gathered data from the California County Mental Health system records regarding women diagnosed with psychiatric disorders. It then compared the birth outcomes of mothers with anxiety disorders, mothers diagnosed with other psychiatric illnesses, and mentally healthy mothers [74]. A data analysis did not yield significant differences among the groups. However, the hypothesis reached statistical significance when comparing African American women with anxiety disorders (SSR equal to 0.92) with African American women free of mental disease (SSR equal to 1.03), implying that African American individuals may deal with more harmful and frequent stressful events in combination with limited support.

Interestingly, in a prospective longitudinal cohort study in the UK, researchers sought to assess the connection between pre-conceptional salivary stress biomarkers and the SSR [75]. Out of 338 women recruited from the Oxford Conception Study, the sample size was limited to 130 delivered singleton births with recorded gender outcomes. The clinical records included salivary cortisol and α-amylase levels. Salivary samples were collected on the sixth day of the first observed menstrual cycle. When comparing the median biomarker concentrations between women delivering male neonates and women delivering female neonates, salivary cortisol was higher, and α-amylase was lower among subjects giving birth to female offspring. However, only cortisol served as a strong predictor of the SSR, with the odds of male birth significantly decreasing among women belonging to the highest quartile of salivary cortisol. These findings indicate that chronic stress prior to conception, expressed as cortisol levels in the saliva, is associated with a reduction in the SSR.

On the other hand, a population-based preconception cohort recruiting 235 couples in Michigan and Texas, USA yielded results opposite to those of prior research [76]. The researchers conducted interviews and determined the stress levels according to Cohen’s Perceived Stress Scale, investigated the potential history of mood and anxiety disorders, and additionally collected maternal saliva samples to measure stress biomarkers. Paternal stress increased the risk of fathering a male neonate by about 76%. The probability of male birth was marginally elevated when records of anxiety disorders were jointly modeled for each couple. Furthermore, the same pattern was observed when ranking the results from lower to higher maternal salivary cortisol concentrations, although no significance was noted. The authors supported that the results were controversial, taking into consideration the established hypothesis that paternal stress around conception decreases testosterone levels, which consequently leads to an excess of female births. Even though not significant, the positive correlation between the SSR and the maternal saliva stress markers opposed previous findings [75]. The authors mentioned that differences may be attributed to the collection of basal saliva samples at different time points compared to the previous study; in the current research, stress markers were measured on the first day following enrollment and at day one of the first observed cycle [76].

A relatively recent study in the USA focused on the influence of prenatal physical stress, apart from psychological status, on the SSR [77]. In total, 187 pregnant women with singleton births were recruited and divided into three groups: healthy, psychologically stressed, and physically stressed. The women who were classified into the psychologically stressed group had been diagnosed with mood and mental health disorders, whereas women belonging to the physically stressed group shared common indicators for physical stress, such as an elevated ambulatory blood pressure. The SSR was significantly lower in the psychologically stressed and physically stressed groups compared to the controls. This was attributed to the increased susceptibility of male embryos to suboptimal conditions in utero due to maternal stress. Of note, the pregnant women who received social support presented higher SSRs.

To sum up, most of the studies come to an agreement that psychological distress provokes a downward shift in the SSR (Figure 1). Specifically, chronic and not acute stress mediates this causal relationship; only cortisol, the primary biomarker of long-lasting stress, which is an end product of the hypothalamus–pituitary–adrenal axis, and not α-amylase, the indicator of acute stress, predicted a decline in the SSR [75]. This decrease in the SSR is speculated to be caused by increased male fetus loss [16]. Another theory explaining the reduced male fetus conception is the less frequent coitus induced by stress, which, especially in the early phase of the menstrual cycle, has been proven to reduce the sex ratio at conception [16,76]. Finally, stress may induce alternations in the SSR through its impact on the sex of the zygote, disrupting sperm’s motility and the female reproductive system [75].

## 6. Occupation and Other Factors

### 6.1. Occupation and SSR

Since a variety of chemicals are speculated to shift the SSR through their impact on the parental reproductive system, the occupational exposure of parents to these substances is a potential contributing factor which can either increase or suppress the index. In this line, a study retrieved data from 1955 to 2005 in Greece and included 587 shipyard employees with 1012 children to examine the SSR in this particular occupation [17]. The SSR among men working as sandblasters/painters and ship carpenters was significantly lower, while the index was not statistically significantly different among fathers in other positions in the shipyard compared to the general population. The authors pointed out that both groups demonstrating the lowest SSR had been systematically exposed to hazardous chemicals, such as solvents, pesticides, polychlorinated biphenyls, and dioxin-like products in wood preservatives and polyaromatic hydrocarbons, which are associated with alterations in the SSR. All of these substances are known for their estrogenic, antiestrogenic, or antiandrogenic activities, causing hormonal imbalances periconceptionally and during pregnancy, thus affecting the SSR.

The interplay of paternal occupational exposure and the SSR was further evaluated in a Japanese study, where the researchers retrieved data from a large-scale birth cohort. They included 50,283 birth outcomes accompanied by information on paternal exposure to different agents around the onset of pregnancy [22]. The SSR among fathers exposed to insecticides was significantly lower than that of the unexposed group. Furthermore, there was a downshift trend in the SSR with an increased exposure to medical disinfectants, such as iodine and ethanol, without reaching statistical significance. The authors mentioned that prior research on insecticides had shown that they are associated with lower semen quality and, thus, reduced fertility. Regarding disinfectants, iodine alters thyroid function and thyroxine levels, which affect semen quality, while ethanol was observed to reduce semen quality in alcoholic men and suppress the SSR in mice. In general, a low semen quality and bicephalic sperm have been associated several times with a downward shift in the SSR [78,79].

Additionally, professional status could be a potential trendsetter of the SSR. A comparative study questioned the latter hypothesis by selecting and studying 353 biographies of women in two volumes from 1921 to 1940 and from 1941 to 1960 in New Zealand [68]. Each biography was studied carefully in order to understand the traits of every subject. It was concluded that high-achieving women give birth to a significantly higher proportion of males compared with the overall SSR. The researchers justified their findings based on the maternal dominance hypothesis, which states that dominant women deliver a higher proportion of male fetuses. This phenomenon is mediated by higher levels of testosterone in these women.

To sum up, the correlation between parental occupation and the SSR represents a multifaceted subject of investigation owing to numerous occupational variables that may impact the offspring ratio (Figure 1). Research indicates variations in the SSR among parents, particularly fathers, who are exposed to specific endocrine-disrupting chemicals. Additionally, occupational standing could influence the SSR, as evidenced by a study linking high-achieving women with an increased likelihood of male offspring.

### 6.2. Other Lifestyle Factors and the SSR

#### 6.2.1. Smoking Habits

Tobacco smoking is well known for its carcinogenic effects on both primary and passive smokers. It is a mixture of at least 4000 compounds, such as cadmium, arsenic, lead, polycyclic aromatic hydrocarbons, carbon monoxide, and formaldehyde. Nicotine, which is an addictive substance, causes vasoconstriction and leads to a lower blood supply in the tissues [80]. Certain compounds, such as cadmium, have been detected in the ovaries and follicular fluid of smokers, which can induce accelerated follicular destruction, chromosomal anomalies in the gametes, and the suppression of estrogen production. Additionally, tobacco smoking has been blamed for adverse birth outcomes, such as miscarriages and lower male and female fertility [80].

A prospective study investigating the effects of smoking and parity on infants’ sexes assessed 2108 singleton births from 1993 to 2002 in Patras, Greece [81]. Subjects who smoked followed that habit during the periconceptional period and throughout pregnancy. The researchers observed that the deliveries of smoking mothers showed a significantly higher SSR compared to the births of nonsmoking mothers. Among the smoking women, parity affected the SSR to a great extent, with the primiparous women delivering significantly more males than females compared to the multiparous mothers. Furthermore, in primiparous smoking women, there was a positive association between the SSR and the number of cigarettes smoked per day. Thus, it was concluded that both smoking status and parity influenced the birth outcome regarding gender. The authors proposed that initially, smoking women have higher estrogen concentrations, which, according to the hormonal hypothesis of James, favors male over female births. However, smoking might suppress the production and tissue response to estrogen, leading to a gradual decline in the SSR ranking from primiparous to multiparous women.

#### 6.2.2. Cultural Beliefs

Consanguinity is the sexual relationship or marriage between people with common biological ancestors, usually up to second cousins. Despite the vox populi, it is a common phenomenon, with prevalence fluctuating from 1% in Western societies to 80% in the Middle East [82]. This profound fluctuation is due to sociocultural reasons, as in Eastern countries, there is a general belief that consanguineous marriage reduces the possibilities of hidden uncertainties in health and financial issues enforcing family solidarity [83]. Regarding genetics, the offspring of consanguineous marriages are more genetically burdened because of the increased incidence of homozygosity for genetic disorders [84]. In India, consanguineous marriages show a high prevalence [85]. Bittles et al. studied the effect of such marriages on the SSR in Kartanika, where the frequency of consanguinity was 33.07%. Surprisingly, the SSR did not differ significantly between consanguineous and non-consanguineous marriages [86].

Furthermore, other cultural perceptions also play a role in influencing the SSR. Eastern Asian countries, for example, have experienced an increased incidence of female feticide due to cultural factors, resulting in an elevated SSR [87]. The widespread use of ultrasound techniques for prenatal sex determination during the 1980s significantly skewed the SSR in Eastern countries, particularly in China, India, and South Korea [88]. For this reason, a retrospective study was conducted in Quebec, Canada to investigate the SSR among the Asian community living there between 1981 and 2004 [89]. From 1981 to 1992, the SSR was significantly higher in some Asian races. The study also revealed that birth order impacted the SSR, with higher ratios being observed among firstborns in some instances. The considerably male-skewed SSR was attributed to the practice of sex selection, which became more prevalent with the emergence of ultrasound imaging in Canada during that time. However, after 1992, a decline in the SSR was noticed, which could be attributed to increased awareness and sensitization among healthcare practitioners in Canada.

## 7. Conclusions

Based on the studies analyzed in the present narrative review, there is compelling evidence that lifestyle factors significantly influence the SSR. Specifically, an increased intake of fatty acids and monosaccharides, adherence to proper nutrition, the attainment of higher educational levels, financial prosperity, and favorable housing conditions have been linked to an elevated SSR. Conversely, factors such as undernutrition, socioeconomic disparities, and psychological distress contribute to a decrease in the SSR. These associations are consistent with the Trivers–Willard hypothesis. Additionally, the correlation between parental occupation and the SSR presents a complex area of study, given the multitude of occupational variables that may impact the SSR. Other elements, including smoking habits and cultural beliefs, may also play a role in trends related to the SSR. Further in-depth research in this realm holds great promise for enhancing our comprehension of the intricate interplay between lifestyle choices and offspring sex ratios, supported by contradictory evidence for certain factors. Future studies could delve more deeply into the mechanisms through which specific lifestyle factors influence the SSR, elucidating the underlying biological pathways involved. Additionally, a broader range of lifestyle factors should be considered in forthcoming research endeavors. Understanding how lifestyle factors shape a population’s SSR could offer valuable insights for designing measures to promote gender balance and reproductive well-being.

## Data Availability

Not applicable.
